# 复合式净化体系结合实时直接分析-串联质谱测定畜禽肉中41种兽药残留

**DOI:** 10.3724/SP.J.1123.2022.11022

**Published:** 2023-07-08

**Authors:** Yingshuang XIE, Bo WANG, Chunni LEI, Lanxia LIU, Huan ZHANG, Xingbin BAI, Zonghong KOU

**Affiliations:** 1.兰州海关技术中心,甘肃兰州730010; 1. Lanzhou Customs Technology Center, Lanzhou 730010, China; 2.白银市食品检验检测中心,甘肃白银730090; 2. Baiyin City Food Inspection and Testing Center, Baiyin 730090, China

**Keywords:** 复合式净化体系, 实时直接分析离子源, 多兽药残留, 畜禽肉, composite purification system, direct analysis in real time ion source, multiple veterinary drug residues, livestock and poultry meat

## Abstract

在食品安全问题屡禁不止的市场环境下,兽药残留带来的食品安全问题以及耐药性传递带来的生物安全问题备受关注。该研究建立了复合式净化体系结合实时直接分析-串联质谱(DART-MS/MS)测定畜禽肉中41种兽药残留的方法。首先采用单标溶液进样的方式优化筛选出最佳定性离子对和定量离子对,再按照欧盟2002/657准则要求对溶剂混合标准溶液和基质混合标准溶液中各兽药化合物的丰度比进行计算,确保DART-MS/MS对41种兽药化合物的准确定性,最后将QuEChERS技术的净化原料丙基乙二胺(PSA)、十八烷基键合硅胶(C18)与多壁碳纳米管相结合,形成复合式净化前处理体系,实现了对畜禽肉样品中物理化学性质差异较大的41种兽药化合物的一步净化。实验以41种兽药化合物的定量离子峰面积为判定指标,考察了DART离子源中关键性参数对其检测的影响。针对41种兽药化合物解离常数(p*K*_a_)的差异及各样品基质的特点,以回收率为判定指标分别对提取溶剂、基质分散溶剂、净化方式等进行了优化。确认了以1.0%甲酸乙腈溶液为提取溶剂,含50 mg PSA、50 mg C18的多壁碳纳米管为净化小柱的样品预处理方式。喹诺酮类、磺胺类、硝基咪唑类38种兽药在2~200 μg/L内线性关系良好,相关系数为0.9979~0.9999,检出限为0.5 μg/kg,定量限为2.0 μg/kg;3种氯霉素类兽药在0.5~20 μg/L内线性关系良好,相关系数为0.9995~0.9997,检出限为0.1 μg/kg,定量限为0.5 μg/kg。鸡肉、猪肉、牛肉、羊肉等不同基质样品中的41种兽药在低、中、高3个加标水平下的加标回收率为80.0%~109.6%,日内精密度为0.3%~6.8%,日间精密度为0.4%~7.0%。通过采用国家标准方法和该研究所建立的方法同时对100批次畜禽肉样品(猪肉、鸡肉、牛肉、羊肉各25批次)以及部分已知阳性样品进行测定,结果表明,3批次猪肉样品中检出磺胺嘧啶(89.2、78.1、105.3 μg/kg), 2批次鸡肉样品中检出沙拉沙星(56.3、102.0 μg/kg),其余样品未检出;两种方法对已知阳性样品的检测结果一致。该方法具有操作快速、简单、灵敏、绿色等优点,适用于畜禽肉中多种兽药残留的同时筛查检测。

## Abstract (en)

In a market environment where food safety problems still occur despite repeated prohibitions, food safety problems caused by veterinary drug residues and biological safety problems caused by the transfer of drug resistance have attracted much attention. Herein, a method based on a compound purification system coupled with direct analysis in real time-tandem mass spectrometry (DART-MS/MS) was established to determine 41 different types of veterinary drug residues in livestock and poultry products. First, a single-standard solution sampling method was used to optimize the selection of the best quasi-molecular ion, two daughter ions, and their cone-hole and collision voltages; qualitative and quantitative ion pairs are composed of a quasi-molecular ion and its corresponding daughter ion. The abundance ratios of the drug compounds in standard solutions of the solvent and matrix mixtures were then calculated according to the requirements of the European Union 2002/657 specification. DART-MS/MS was subsequently developed for the accurate characterization and quantitative analysis of the veterinary drugs. Finally, a composite purification pretreatment system was formed by combining the primary secondary amine (PSA) and octadecyl bonded silica gel (C18) of a QuEChERS technology with multiwalled carbon nanotubes (MWCNTs) to achieve the one-step purification of the drug compounds. The influence of the key parameters of the DART ion source on the determination of the drugs was investigated using the peak areas of the quantitative ions as the criterion. The optimum conditions were as follows: ion source temperature of 350 ℃, 12-Dip-it Samplers module, sample injection speed of 0.6 mm/s, and external vacuum pump pressure of -75 kPa. According to the differences in the dissociation constant (p*K*_a_) ranges of the 41 types of veterinary drug compounds and the characteristics of the sample matrixes, the extraction solvent, matrix-dispersing solvent, and purification method were optimized based on the recovery. The extraction solvent was 1.0% acetonitrile formate solution, and the pretreatment column included MWCNTs containing 50 mg of PSA and 50 mg of C18. The three chloramphenicol drugs showed a linear relationship in the ranges of 0.5-20 μg/L with correlation coefficients of 0.9995-0.9997,and the detection and quantification limits of three chloramphenicol drugs were 0.1 and 0.5 μg/kg, respectively. The 38 other drugs, including quinolones, sulfonamides, and nitro-imidazoles showed a linear relationship in the ranges of 2-200 μg/L with correlation coefficients of 0.9979-0.9999, and the detection and quantification limits of the 38 other drugs were 0.5 and 2.0 μg/kg, respectively. The recoveries of the 41 veterinary drugs at low, medium, and high spiked levels in chicken, pork, beef, and mutton samples were 80.0%-109.6%, with intra- and inter-day precisions of 0.3%-6.8% and 0.4%-7.0%, respectively. A total of 100 batches of animal meat (pork, chicken, beef, and mutton; 25 batches each) and known positive samples were simultaneously analyzed using the national standard method and the detection method established in this study. Sulfadiazine (89.2, 78.1, and 105.3 μg/kg) was detected in three batches of pork samples, and sarafloxacin (56.3, 102.0 μg/kg) was detected in two batches of chicken samples and no veterinary drugs were detected in the other samples; both methods yielded consistent results for known positive samples. The proposed method is rapid, simple, sensitive, environmentally friendly, and suitable for the simultaneous screening and detection of multiple veterinary drug residues in animal meat.

畜禽肉作为人们重要的食物来源,已成为我国进出口贸易中最受关注的商品。据海关数据统计,2021年间我国畜禽肉的进出口数量为57984万千克,总贸易金额高达154180万元^[1]^。随着进出口贸易额的增加,畜禽肉的质量安全问题越来越受到关注。不科学的养殖行为及经济利益的驱使导致畜禽肉食品中兽药残留超标的现象时有发生^[2]^,其中以广谱性强、临床效果显著、抑菌活性较强的氯霉素类、喹诺酮类、磺胺类、硝基咪唑类等药物引起的兽药残留问题较为严重^[3]^。一直以来,国际食品法典委员会、欧盟、美国食品药品监督管理局等都对兽药的使用进行了限制。我国的《食品安全国家标准 食品中兽药最大残留限量》(GB 31650-2019)^[4]^和中华人民共和国农业农村部公告第250号^[5]^等规范中对氯霉素类、喹诺酮类、磺胺类、硝基咪唑类等药物的限量进行了严格规定。相继出台的兽药残留检测标准以分类检测各样品基质和兽药为主,存在样品预处理复杂、不同种类基质和药物检测耗时较长的问题。因此,建立一个能够同时准确测定畜禽肉中多种兽药残留的快速筛查方法非常必要,这对畜禽肉中兽药残留的大批量检测和提高监管效率具有重要的意义。

畜禽肉中兽药残留检测的样品前处理方法主要有液液萃取法(LLE)^[6]^、QuEChERS法^[7]^、固相萃取法(SPE)^[8,9]^等。这些前处理方法存在操作繁琐、试剂消耗量大、前处理时间较长、净化不充分、净化填料粒径较小而易在样液中残留从而产生基质效应等问题。本研究拟采用的净化体系是将QuEChERS原理结合固相萃取柱的柱填充理念形成一步净化柱,与饱和正己烷的二次除酯技术共同组成的复合式净化体系。该净化体系简化了SPE和QuEChERS中的操作步骤,有效提高了检测效率。

实时直接分析-串联质谱技术(DART-MS/MS)采用氦激发态电离实现目标化合物的离子化,其不易受蛋白质、脂肪、盐等影响而产生基质效应,不易产生加合离子,不需要色谱分离即可实现检测^[10,11]^。该技术广泛应用于农药多残留分析,但在畜禽肉样品中同时测定氯霉素类、磺胺类、喹诺酮类、硝基咪唑类药物鲜有报道。本研究拟采用一步净化前处理技术与DART-MS/MS相结合对畜禽肉中的41种兽药残留进行快速检测,为畜禽肉中多种兽药快速检测方法的建立以及快速检测标准的制定提供新的研究思路。

## 1 实验部分

### 1.1 仪器、试剂与材料

DART离子源(美国Ion-Sense公司); 6460A三重四极杆串联质谱仪(美国安捷伦公司);电子分析天平(上海梅特勒-托利多仪器有限公司);多管式涡旋振荡器(德国Heidolph公司); 3K30低温冷冻高速离心机(德国Sigma-Aldrich公司);氮吹仪(美国Organomation公司); 1~10 μL、10~100 μL、100~1000 μL移液枪(Eppendorf中国有限公司)。

41种兽药标准溶液:9种喹诺酮类单标溶液、15种磺胺类单标溶液、3种氯霉素类单标溶液、14种硝基咪唑类单标溶液,质量浓度均为100 mg/L,溶剂均为甲醇(上海安谱实验科技股份有限公司)。

8种内标溶液:喹诺酮类(诺氟沙星-D5、环丙沙星-D8、恩诺沙星-D5),磺胺类(磺胺邻二甲氧嘧啶-D3、磺胺间二甲氧基嘧啶-D6),氯霉素类(氯霉素-D5),硝基咪唑类(甲硝唑-D3、替硝唑-D5),质量浓度均为100 mg/L(上海安谱实验科技股份有限公司)。

甲醇、乙腈、乙酸乙酯、正己烷、甲酸(色谱纯,德国Merck公司);乙二胺四乙酸二钠(EDTA,纯度≥99.0%)、无水硫酸镁(纯度≥99.0%)、无水硫酸钠(纯度≥99.8%)(国药集团化学试剂有限公司);十八烷基键合硅胶(C18)、丙基乙二胺(PSA)(美国Agilent公司);屈臣氏超纯水(屈臣氏集团有限责任公司);多壁碳纳米管、防水隔膜(北京绿绵有限责任公司); HLB固相萃取柱(500 mg/6 mL,沃特世上海有限责任公司)。

### 1.2 标准溶液的配制

氯霉素类混合标准储备液:准确吸取100 μL氯霉素、甲砜霉素、氟苯尼考单标溶液于10 mL容量瓶中,用甲醇稀释、定容,配制成质量浓度为1 mg/L的氯霉素类混合标准储备液,置于-18 ℃保存。

混合标准溶液:分别准确吸取100 μL喹诺酮类、磺胺类、硝基咪唑类单标溶液和1 mL 3种氯霉素类混合标准储备液于10 mL容量瓶中,用甲醇稀释、定容,配制成质量浓度为1 mg/L(3种氯霉素类化合物的质量浓度为0.1 mg/L)的混合标准溶液,置于-18 ℃保存。

混合内标标准溶液:准确吸取100 μL上述喹诺酮类、磺胺类、硝基咪唑类内标溶液和10 μL氯霉素类内标溶液于10 mL容量瓶中,用甲醇稀释、定容,配制成混合内标标准储备液(喹诺酮类、磺胺类、硝基咪唑类、氯霉素类内标的质量浓度分别为1、1、1、0.1 mg/L),置于-18 ℃保存。

混合基质标准溶液:在900 μL经样品前处理所得的空白基质溶液中加入100 μL混合标准溶液,得到质量浓度为100 μg/L(3种氯霉素类化合物的质量浓度为10 μg/L)的混合基质标准溶液,现用现配。

### 1.3 样品前处理

精确称取均质后的样品2.00 g(精确至0.01 g)于带有两粒陶瓷均质子的50 mL具塞离心管中,依次加入0.1 mL的混合内标标准溶液、0.1 mol/L EDTA缓冲溶液,涡旋振荡3 min,使畜禽肉样品充分分散混匀;之后加入10 mL 1.0%甲酸乙腈溶液,涡旋振荡10 min后,依次加入4 g无水硫酸镁、1 g氯化钠涡旋振荡1 min,于4 ℃以13000 r/min高速冷冻离心5 min,移取上清液于另一50 mL离心管中,待净化。

取一步净化柱(含50 mg PSA、50 mg C18的多壁碳纳米管组成的净化小柱),将带有填料的一端垂直塞入含有待净化液的50 mL具塞离心管内,缓慢下压净化小柱,直至小柱内的待测液体积达到5 mL以上。取5 mL待测液至15 mL离心管中,于40 ℃水浴氮吹至近干;加入5 mL饱和正己烷(将30%乙腈正己烷溶液涡旋振荡5 min后静置分层,上层即为饱和正己烷),涡旋3 min,于4000 r/min离心3 min,弃去饱和正己烷;准确加入1 mL 80%乙腈水溶液复溶,涡旋混合1 min,过0.22 μm有机相滤膜于进样瓶中,待上机分析。

### 1.4 仪器条件

离子源:DART离子源;采集模式:正、负离子化模式;离子化气体:氦气;离子源温度:350 ℃;进样模块:12-Dip-it Samplers模块;进样速度:0.6 mm/s;外置真空泵的真空度:-75 kPa。MS/MS参数条件:毛细管电压3500 kV,干燥气温度300 ℃,干燥气流量1 L/min,雾化气压力13.8 kPa (2 psi),采集模式为多反应监测(MRM)模式,经过优化后的41种兽药及8种内标的质谱参数见表1。

**表1:** 41种兽药及8种内标的质谱参数

No.	Compound	Parent ion (*m/z*)	Daughter ions (*m/z*)	DP/ V	CEs/ eV	Ion source
1	sulfamethazine (磺胺二甲嘧啶)	279.1	186.0^*^,124.0	110	15,20	DART^+^
2	sulfamethoxazole (磺胺甲基异恶唑)	254.1	108.1^*^,92.1	110	35,35	DART^+^
3	sulfamonomethoxine (磺胺-6-甲氧嘧啶)	281.0	156.0^*^,92.0	110	22,35	DART^+^
4	sulfaquinoxaline (磺胺喹恶啉)	301.1	156.0^*^,92.0	130	16,30	DART^+^
5	sulfadimethoxine (磺胺二甲氧嘧啶)	311.0	156.0^*^,92.0	130	20,32	DART^+^
6	trimethoprim (甲氧苄啶)	291.3	123.0^*^,230.2	125	30,30	DART^+^
7	sulfapyridine (磺胺吡啶)	250.0	92.1^*^,156.1	100	32,15	DART^+^
8	sulfadiazine (磺胺嘧啶)	251.0	156.0^*^,92.0	115	15,27	DART^+^
9	sulfamethoxazole (磺胺甲恶唑)	254.1	92.1^*^,108.1	110	30,30	DART^+^
10	sulfathiazole (磺胺噻唑)	256.0	156.1^*^,92.1	100	30,15	DART^+^
11	sulfamerazine (磺胺甲基嘧啶)	265.1	156.1^*^,65.1	100	10,40	DART^+^
12	sulfamoxol (磺胺二甲恶唑)	285.1	156.2^*^,108.0	100	23,33	DART^+^
13	sulfisoxazole (磺胺异恶唑)	268.1	156.0^*^,113.0	115	30,13	DART^+^
14	sulfamethoxypyridazine (磺胺甲氧哒嗪)	285.1	108.0^*^,156.0	120	22,10	DART^+^
15	sulfamonomethoxine (磺胺间甲氧嘧啶)	281.0	156.1^*^,65.1	100	20,30	DART^+^
16	norfloxacin (诺氟沙星)	320.2	302.2^*^,276.2	115	15,13	DART^+^
17	ofloxacin (氧氟沙星)	362.2	318.2^*^,261.1	135	10,30	DART^+^
18	pefloxacin (培氟沙星)	334.2	316.2^*^,70.1	115	15,45	DART^+^
19	ciprofloxacin (环丙沙星)	332.3	288.2^*^,245.2	125	15,25	DART^+^
20	enrofloxacin (恩诺沙星)	360.2	342.1^*^,316.1	135	10,10	DART^+^
21	danofloxacin (达氟沙星)	358.0	314.1^*^,340.1	135	20,25	DART^+^
22	sarafloxacin (沙拉沙星)	386.3	342.2^*^,299.2	140	20,30	DART^+^
23	difloxacin (二氟沙星)	400.3	356.3^*^,299.2	135	20,20	DART^+^
24	enoxacin (依诺沙星)	321.3	303.1^*^,232.1	120	10,30	DART^+^
25	pimonidazole (哌莫硝唑)	255.0	124.3^*^,98.3	110	20,15	DART^+^
26	hydroxymetronidazole (羟基甲硝唑)	187.7	126.1^*^,123.1	80	25,30	DART^+^
27	1-methyl-5-nitro-1*H*-imidazole-2-methanol (羟基二甲硝咪唑)	157.9	94.3^*^,55.4	80	25,25	DART^+^
28	metronidazole (甲硝唑)	172.3	127.9^*^,82.1	80	10,25	DART^+^
29	tinidazole (替硝唑)	247.8	128.2^*^,82.3	90	25,20	DART^+^
30	2-dimethyl-5-nitro-1*H*-imidazole-1-ethanol (塞克硝唑)	186.1	128.2^*^,82.3	90	20,25	DART^+^
31	2-methyl-5-nitro-1*H*-imidazole-1-propanolmonohydrochloride (特尼达唑)	186.2	128.2^*^,82.3	90	20,25	DART^+^
32	hydroxy-ipronidazole (羟基异丙硝唑)	185.9	168.2^*^,122.3	90	20,20	DART^+^
33	ipronidazole (异丙硝唑)	169.8	124.1^*^,109.1	85	20,20	DART^+^
34	benznidazole (苄硝唑)	261.0	107.3^*^,91.3	110	15,25	DART^+^
35	fexinidazole (非昔硝唑)	233.1	93.3^*^,106.3	110	15,25	DART^+^
36	panidazole (帕硝唑)	233.0	106.3^*^,93.3	110	25,10	DART^+^
37	carnidazole (卡硝唑)	245.0	118.2^*^,75.2	110	20,10	DART^+^
38	misonidazole (米索硝唑)	202.1	145.3^*^,174.2	110	25,15	DART^+^
39	chloramphenicol (氯霉素)	321.0	152.0^*^,257.1	110	11,4	DART^-^
40	florfenicol (氟苯尼考)	247.9	229.8^*^,130.1	110	10,22	DART^-^
41	thiamphenicol (甲砜霉素)	354.1	185.1^*^,290.1	110	17,5	DART^-^
42	norfloxacin-D5 (诺氟沙星-D5)	325.0	306.9^*^,280.9	100	15,15	DART^+^
43	ciprofloxacin-D8 (环丙沙星-D8)	340.1	322.1^*^,234.8	80	20,40	DART^+^
44	enrofloxacin-D5 (恩诺沙星-D5)	365.3	347.2^*^,321.3	125	19,15	DART^+^
45	sulfadoxine-D3 (磺胺邻二甲氧嘧啶-D3)	314.0	156^*^	125	17	DART^+^
46	sulfadimethoxine-D6 (磺胺间二甲氧基嘧啶-D6)	317.0	156^*^	125	20	DART^+^
47	metronidazole-D3 (甲硝唑-D3)	175.1	127.9^*^	80	10	DART^+^
48	chloramphenicol-D5 (氯霉素-D5)	325.8	156.6^*^	130	15	DART^-^
49	tinidazole-D5 (替硝唑-D5)	253.1	126.1^*^	110	15	DART^+^

DP: declustering potential; CE collision energy; * quantitative ion.

## 2 结果与讨论

### 2.1 质谱条件的优化

#### 2.1.1 定性指标分析

DART-MS/MS技术在对目标化合物进行分析时不需要进行色谱分离,为防止假阳性,定性指标的确定尤为重要。按照欧盟2002/657准则要求,质谱方法是通过化合物的离子丰度比来对化合物进行最终定性的^[12,13]^。首先,本实验采用单标溶液进样的方式优化筛选出最佳准分子离子和2个子离子及其对应的最佳锥孔电压和碰撞能量,组成1个准分子离子和1个子离子一一对应的定性离子对。其次,基于化合物在固定碰撞能量下的碎裂方式及形成碎片信息的稳定性,即溶剂中目标化合物的离子丰度比与基质中目标化合物的离子丰度比一致的原则^[16⇓-18]^,采用本方法对阴性畜禽肉样品进行预处理得到空白基质溶液,将空白基质溶液配制成质量浓度为100 μg/L(3种氯霉素类化合物的质量浓度为10 μg/L)的混合基质标准溶液,经仪器平行测定12次后,计算其定性离子对丰度比的平均值,结果表明其平均值符合欧盟2002/657准则对相对离子丰度的要求^[12,13]^,相关要求见表2。因此本研究可实现多种兽药检测的准确定性。

**表2:** 质谱技术中相对离子丰度的最大允许偏差范围

*K*	Maximum allowable deviation range/%
*K*>50	±20
20<*K*≤50	±25
10<*K*≤20	±30
*K*≤10	±50

#### 2.1.2 离子源温度的优化

对于DART离子源,离子源温度是影响其灵敏度的关键性因素,目标化合物的离子化效率会随着温度的改变而受到影响。配制100 μg/L(3种氯霉素类化合物的质量浓度为10 μg/L)混合基质标准溶液,分别采用150、200、250、300、350、400、450、500 ℃的离子源温度对其进行分析,每个温度下进行6次平行实验,利用氯霉素类、喹诺酮类、磺胺类、硝基咪唑类4类兽药峰面积的平均值作为判定指标来对离子源温度进行优化,结果见图1。离子源温度为350 ℃时,4类兽药的平均响应值最高,说明其离子化效率和灵敏度最高;离子源温度≥400 ℃时,带有样品的12-Dip-it尖头有轻微焦化现象,4类兽药的离子化效率降低,说明温度过高基质样品会轻微焦化而引入杂质离子,从而影响目标化合物的离子化效率^[14,15]^。因此,将离子源温度设定为350 ℃。

**图1:**
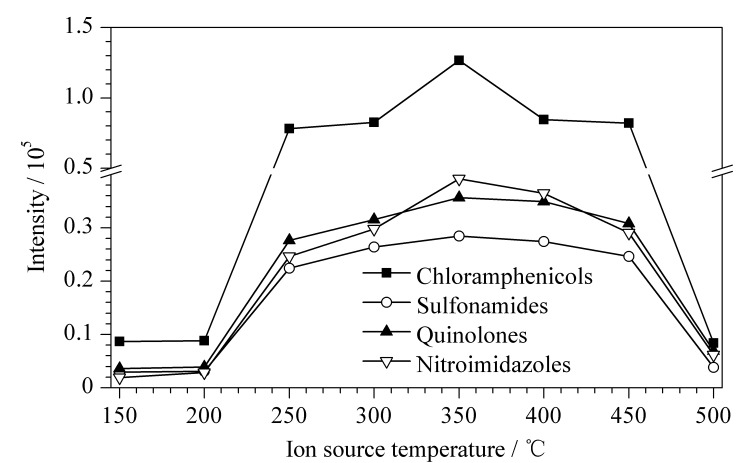
不同离子源温度下4类兽药的平均响应值

#### 2.1.3 进样模块的选择

进样模块对DART离子源的样品间交叉污染、采样平行性、定量准确性等有直接影响。目前DART常见的进样模块有筛网和12-Dip-it Samplers两种。12-Dip-it Samplers进样模块易清洗,不易形成样品间的交叉污染,成本较低,更适用于基质复杂的畜禽肉样品检测需求,但其存在平行性较差、定量不准确的问题。本研究通过实验证明,采用内标加入法可对色谱峰形进行校正,解决样品间平行性较差的问题,从而实现准确定量。因此选择12-Dip-it Samplers作为进样模块,采用内标法定量。

#### 2.1.4 进样速度的优化

对于DART离子源,随着进样速度的增加,样品在离子化区域停留的时间缩短,灵敏度下降;进样速度减慢易引起峰展宽、峰拖尾、双峰等现象,灵敏度下降且影响定性分析^[16]^。本实验分别以0.2、0.4、0.6、0.8 mm/s的进样速度进行优化。由图2可知,随着进样速度的增加,4类兽药的平均响应值增加,其离子化效率也逐渐增高;当进样速度为0.6 mm/s时,4类兽药的平均响应值最高。因此本研究最终选择0.6 mm/s作为分析41种兽药的进样速度。

**图2:**
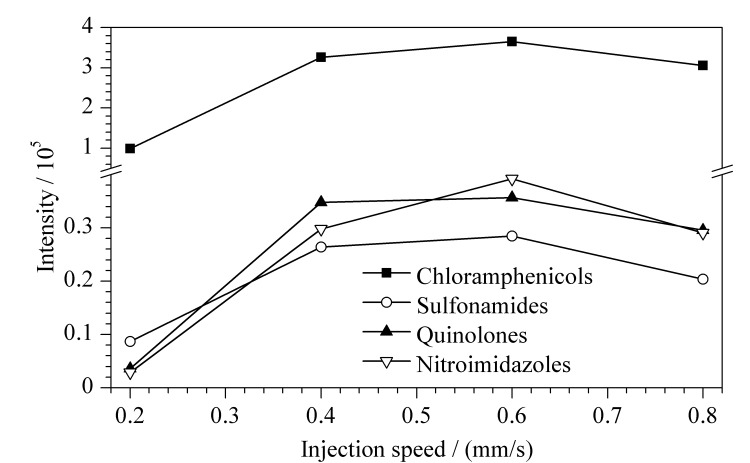
不同进样速度下4类兽药的平均响应值

#### 2.1.5 外置真空泵真空度的优化

DART是开放式离子源,为保证质谱的真空度以及目标化合物的采集量,需要配置外置真空泵^[17]^。本实验对外置真空泵的真空度(-60、-65、-70、-75、-80、-90 kPa)进行了优化,结果见图3。结果表明,随着真空度的增加,4类兽药的平均响应值逐渐增加,说明其灵敏度逐渐增加,当真空度达到-75 kPa时趋于稳定,-80、-90 kPa时变化较小。因此,最终选择-75 kPa为分析41种兽药的外置泵真空度。

**图3:**
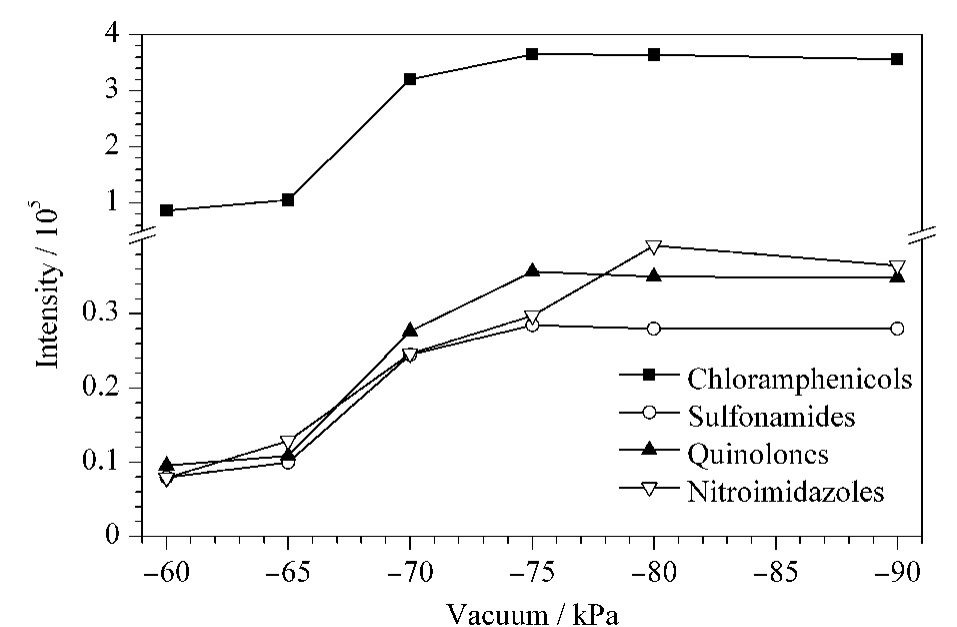
不同真空度下4类兽药的平均响应值

### 2.2 基质分散溶剂的优化

畜禽肉样品在乙腈溶液中易结团成块,影响目标化合物的提取。在对该类样品进行检测时,会先用水溶液将畜禽肉样品溶解使其分散^[19,20]^。本实验考察了纯水、EDTA缓冲溶液对畜禽肉样品的分散效果。结果表明,EDTA缓冲溶液可有效分散畜禽肉样品,防止金属离子与蛋白质结合,使喹诺酮类化合物游离出来。因此本研究选择EDTA缓冲溶液作为基质分散溶剂。

### 2.3 提取溶剂的优化

本实验以4类兽药的平均回收率(*n*=6)为指标,分别考察了甲醇、乙酸乙酯和乙腈的提取效果。结果表明,甲醇的极性较强,实验过程中提取的杂质较多,会影响目标化合物的离子化效率,4类兽药的平均回收率偏低,为50%~70%。乙酸乙酯在实验过程中易提取大量油脂,净化过程中易造成柱堵塞,影响目标物的回收率,其中喹诺酮类化合物的回收率为20%~35%。由于乙腈的样品组织穿透力强,能够较好地沉淀畜禽肉样品中的脂肪和蛋白质,在用乙腈作为提取溶剂时,4类兽药的平均回收率可达80.56%~106.0%。

为抑制酸性或碱性化合物的解离,兽药残留的提取过程中大多用氨水、有机酸等调节体系的pH值,从而改变分配系数,增加兽药在乙腈溶液中的分配比例。本研究分别以0.1%氨水乙腈和不同体积分数(0.1%、0.5%、1.0%、1.5%、2.0%)的甲酸乙腈溶液为提取溶剂进行考察。结果表明,在碱性提取体系中,硝基咪唑类化合物的平均回收率大于80%,喹诺酮类化合物的平均回收率较差,只有20%;在酸性提取体系中,随着甲酸体积分数的增加,4类兽药的平均回收率逐渐增加,采用1.0%甲酸乙腈时4类兽药的平均回收率最高;当甲酸的体积分数达到1.5%时,4类兽药的平均回收率开始下降,结果如图4所示。卞华等^[18]^的研究表明,过高体积分数的甲酸会引起畜禽肉中蛋白质变性,增加样品中的胶体物质含量,从而影响回收率。因此本研究选择1.0%甲酸乙腈作为提取溶剂。

**图4:**
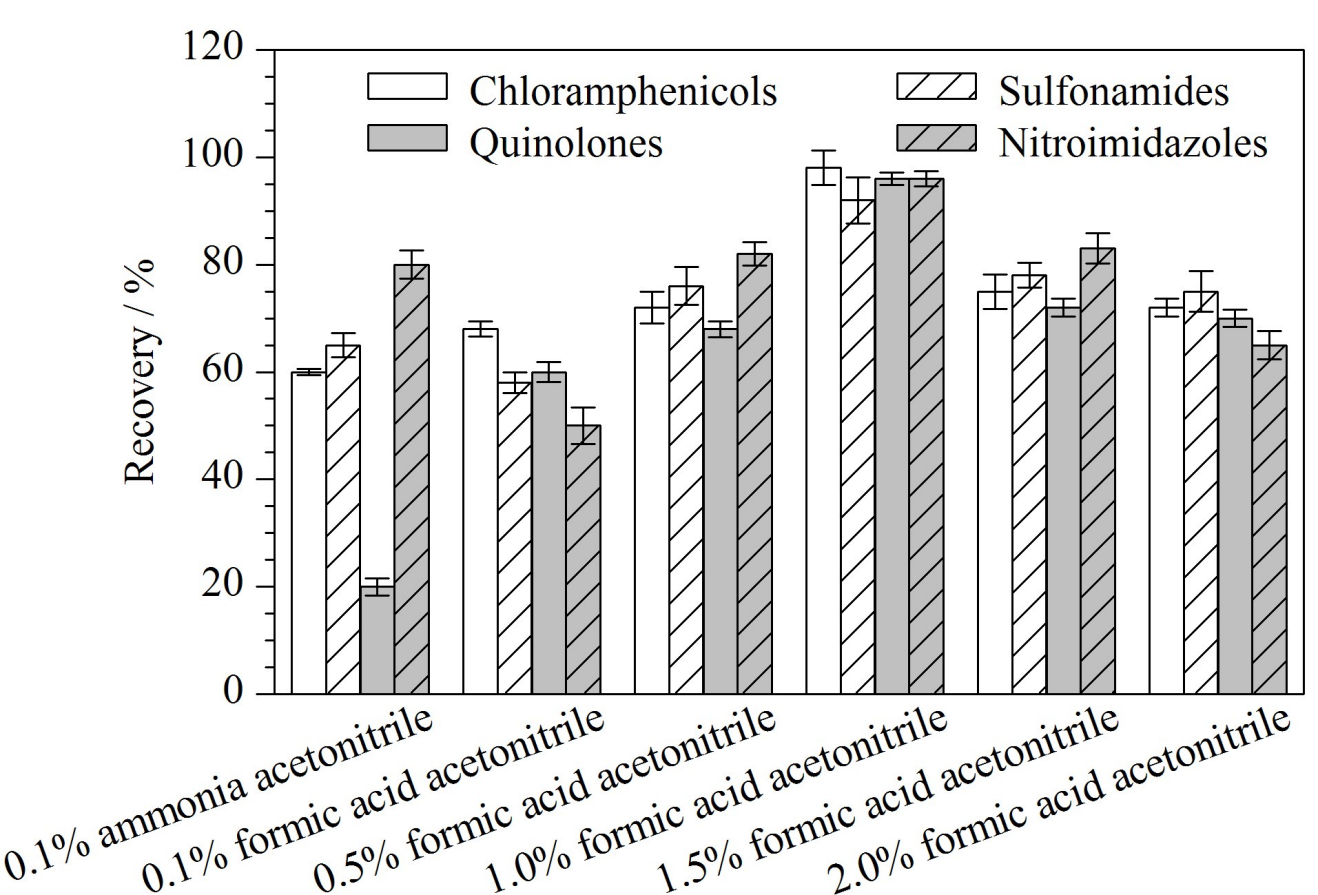
不同提取溶剂对4类兽药平均回收率的影响(*n*=6)

### 2.4 净化方式的优化

畜禽肉样品基质较为复杂,脂肪、蛋白质含量较高^[21]^。本实验考察了HLB固相萃取法、QuEChERS法、一步净化柱法(含50 mg PSA、50 mg C18的多壁碳纳米管组成的净化小柱)对畜禽肉中41种兽药的净化效果。结果表明,41种兽药在3种净化方式下的回收率均较为稳定。相比于QuEChERS法和HLB固相萃取法,一步净化柱无需重复涡旋、离心、活化、上样、淋洗、洗脱等过程;同时将净化液氮吹至近干后,采用饱和正己烷进一步去除油脂,以提高回收率。因此,本实验建立的复合式净化体系能有效去除畜禽肉样品中的蛋白质、磷脂、脂肪等杂质,具有操作简单、耗时较短等优点。

### 2.5 方法学评估

#### 2.5.1 线性范围、方法检出限和定量限

分别称取鸡肉、猪肉、牛肉、羊肉样品各7份,其中1份做空白实验,其余6份准确加入0.1 mL 1.2节中的混合内标标准溶液,依次加入1.2节中的混合标准溶液0.004、0.010、0.100、0.150、0.200、0.400 mL,按照1.3节样品前处理方法进行处理。以41种兽药的质量浓度为横坐标(*x*), 41种兽药与对应内标的峰面积之比为纵坐标(*y*)绘制线性曲线,41种兽药在猪肉基质中的相关数据见表3。目标化合物与内标物质的对应关系如下:氧氟沙星、培氟沙星、依诺沙星、诺氟沙星以诺氟沙星-D5为内标;二氟沙星、环丙沙星、达氟沙星以环丙沙星-D8为内标;恩诺沙星、依诺沙星、沙拉沙星以恩诺沙星-D5为内标;磺胺二甲嘧啶、磺胺甲基异恶唑、磺胺-6-甲氧嘧啶、磺胺二甲氧嘧啶、磺胺吡啶、磺胺嘧啶、磺胺甲恶唑、磺胺噻唑、磺胺甲基嘧啶、磺胺二甲恶唑、磺胺异恶唑、磺胺间甲氧嘧啶以磺胺邻二甲氧嘧啶-D3为内标;磺胺喹恶林、甲氧苄啶、磺胺甲氧哒嗪以磺胺间二甲氧基嘧啶-D6为内标;氟苯尼考、甲砜霉素、氯霉素是以氯霉素-D5为内标;羟基甲硝唑、羟基二甲硝咪唑、甲硝唑、赛克硝唑、特尼哒唑、米索硝唑以甲硝唑-D3为内标;替硝唑、羟基异丙硝唑、异丙硝唑、苄硝唑、非昔硝唑、帕硝唑、卡硝唑、哌莫硝唑以替硝唑-D5为内标。

**表3:** 41种兽药在猪肉基质中的线性范围、相关系数、检出限和定量限(*n*=6)

No.	Compound	Linear range/ (μg/L)	*r*	LOD/ (μg/kg)	LOQ/ (μg/kg)	No.	Compound	Linear range/ (μg/L)	*r*	LOD/ (μg/kg)	LOQ/ (μg/kg)
1	sulfadimidine	2-200	0.9993	0.5	2.0	24	enoxacin	2-200	0.9998	0.5	2.0
2	sulfamethoxazole	2-200	0.9998	0.5	2.0	25	garms nitrous oxide	2-200	0.9995	0.5	2.0
3	sulfamonomethoxine	2-200	0.9995	0.5	2.0	26	hydroxymetronidazole	2-200	0.9998	0.5	2.0
4	sulfaquinoxaline	2-200	0.9998	0.5	2.0	27	1-methyl-5-nitro-1*H*-	2-200	0.9996	0.5	2.0
5	sulfadimethoxine	2-200	0.9997	0.5	2.0		imidazole-2-methanol				
6	trimethoprim	2-200	0.9998	0.5	2.0	28	metronidazole	2-200	0.9998	0.5	2.0
7	sulfapyridine	2-200	0.9979	0.5	2.0	29	tinidazole	2-200	0.9996	0.5	2.0
8	sulfadiazine	2-200	0.9990	0.5	2.0	30	2-dimethyl-5-nitro-1*H*-	2-200	0.9998	0.5	2.0
9	sulfamethoxazole	2-200	0.9999	0.5	2.0		imidazole-1-ethanol				
10	sulfathiazole	2-200	0.9997	0.5	2.0	31	2-methyl-5-nitro-1*H*-	2-200	0.9996	0.5	2.0
11	sulfamethyldiazine	2-200	0.9998	0.5	2.0		imidazole-1-propanol				
12	sulfamethazole	2-200	0.9998	0.5	2.0		monohydrochloride				
13	sulfisoxazole	2-200	0.9987	0.5	2.0	32	hydroxy-ipronidazole	2-200	0.9998	0.5	2.0
14	sulfamethoxypyridazine	2-200	0.9994	0.5	2.0	33	ipronidazole	2-200	0.9997	0.5	2.0
15	sulfamonomethoxine	2-200	0.9997	0.5	2.0	34	benznidazole	2-200	0.9998	0.5	2.0
16	norfloxacin	2-200	0.9998	0.5	2.0	35	fexinidazole	2-200	0.9995	0.5	2.0
17	ofloxacin	2-200	0.9996	0.5	2.0	36	panidazole	2-200	0.9998	0.5	2.0
18	peflacine	2-200	0.9995	0.5	2.0	37	carnidazole	2-200	0.9996	0.5	2.0
19	ciprofloxacin	2-200	0.9999	0.5	2.0	38	misonidazole	2-200	0.9998	0.5	2.0
20	ennosault	2-200	0.9999	0.5	2.0	39	chloramphenicol	0.5-20	0.9995	0.1	0.5
21	danofloxacin	2-200	0.9996	0.5	2.0	40	florfenicol	0.5-20	0.9997	0.1	0.5
22	sarafloxacin	2-200	0.9998	0.5	2.0	41	thiamphenicol	0.5-20	0.9996	0.1	0.5
23	difloxacin	2-200	0.9996	0.5	2.0						

由表3可以看出,氯霉素、氟苯尼考、甲砜霉素在0.5~20 μg/L内线性关系良好,相关系数为0.9995~0.9997;其余38种化合物在2~200 μg/L内线性关系良好,相关系数为0.9979~0.9999。

本实验以3倍信噪比(*S/N*≥3)确定方法检出限,以10倍信噪比(*S/N*≥10)确定方法定量限。结果如表3所示,3种氯霉素类化合物的检出限为0.1 μg/kg,定量限为0.5 μg/kg;其余38种化合物的检出限为0.5 μg/kg,定量限为2.0 μg/kg。

#### 2.5.2 回收率和精密度

分别称取鸡肉、猪肉、牛肉、羊肉阴性样品各18份,按照41种兽药方法定量限的1、5、10倍添加混合标准溶液,然后准确加入0.1 mL 1.2节中的混合内标标准溶液,按照1.3节方法进行样品前处理,并进行仪器检测,内标法定量,计算各基质样品的加标回收率和精密度,其中猪肉基质的加标回收率和精密度列于表4,其余样品基质(鸡肉、牛肉、羊肉)的实验结果见附表1~3(www.chrom-China.com)。实验结果表面,41种兽药在所测畜禽肉样品中的加标回收率为80.0% ~109.6%,精密度为0.8%~7.1%。将上述3个加标样品预处理后的样液每间隔4 h各平行采样6次(一日内共采样6次,*n*=6),计算日内精密度;在同一时间点连续采样7 d,每天平行采样6次(*n=*7),计算日间精密度;结果见表4。结果表明,41种兽药的日内精密度为0*.*3*%~*6*.*8*%*,日间精密度为0*.*4*%~*7*.*0*%*。实验结果说明,该方法的准确性和精密度均较为理想,能够达到多类别兽药的定性、定量检测要求。

**表4:** 猪肉样品中41种兽药在不同加标水平下的回收率和精密度(*n*=6)

Compound	2.0 μg/kg										10.0 μg/kg										20.0 μg/kg							
	Recovery/ %		RSD/ %		Intra- day RSD/%		Inter- day RSD/%^#^				Recovery/ %		RSD/ %		Intra- day RSD/%		Inter- day RSD/%^#^				Recovery/ %		RSD/ %		Intra- day RSD/%		Inter- day RSD/%^#^	
Sulfadimidine		82.4		5.7		4.3		2.3				87.5		2.4		2.1		3.3				82.6		3.2		3.6		3.5
Sulfamethoxazole		93.2		5.9		2.8		2.9				90.2		1.5		3.2		3.5				98.2		4.1		2.9		3.4
Sulfamonomethoxine		97.0		4.8		1.5		3.1				85.8		4.5		3.1		3.8				82.6		4.1		3.5		2.8
Sulfaquinoxaline		93.2		4.5		4.1		2.9				84.2		3.6		2.6		3.2				85.6		3.2		2.9		4.2
Sulfadimethoxine		95.6		3.7		3.2		4.2				88.5		4.5		2.6		3.2				95.2		2.1		2.4		3.1
Trimethoprim		86.5		4.0		3.2		6.3				98.6		2.8		3.2		4.1				93.6		3.5		3.5		3.9
Sulfapyridine		88.0		3.8		4.2		3.6				81.1		4.8		2.8		5.4				89.9		2.5		4.6		3.0
Sulfadiazine		89.4		3.2		3.5		3.9				88.9		2.7		3.8		3.9				96.3		3.6		6.2		2.9
Sulfamethoxazole		92.6		3.3		3.1		3.6				91.9		4.3		1.8		4.3				97.2		3.8		4.1		6.8
Sulfathiazole		83.7		4.2		3.9		2.3				80.6		2.4		1.7		4.4				87.9		1.3		2.4		6.6
Sulfamethyldiazine		85.8		6.9		1.9		1.3				90.2		5.6		1.1		3.2				87.1		3.1		2.4		2.5
Sulfamethazole		90.8		6.3		2.3		4.9				85.4		3.8		0.9		5.2				106.0		3.6		2.2		5.1
Sulfisoxazole		100.2		3.5		1.1		2.3				92.6		4.6		1.6		3.8				95.6		4.4		4.1		3.3
Sulfamethoxypyridazine		87.9		5.2		0.9		3.2				96.2		3.2		3.4		6.6				95.6		3.6		2.6		2.1
Sulfamonomethoxine		94.5		2.8		1.1		4.8				84.7		1.2		2.9		4.2				99.2		2.8		4.6		3.3
Norfloxacin		96.3		6.5		2.6		2.5				88.8		2.9		4.6		5.3				85.4		3.6		3.3		3.3
Ofloxacin		89.8		3.9		4.9		3.2				97.1		2.2		2.6		3.5				99.5		3.9		3.2		4.1
Peflacine		84.9		4.2		5.6		2.7				85.9		2.4		6.3		2.2				102.0		4.2		2.6		3.7
Ciprofloxacin		90.0		1.2		3.1		1.9				90.6		1.5		3.9		2.2				85.6		2.6		3.6		2.9
Ennosault		97.4		3.9		6.2		3.6				88.1		4.5		5.2		3.2				98.2		2.9		2.9		2.6
Danofloxacin		96.8		2.8		4.2		4.1				96.7		5.6		3.3		2.5				101.0		3.2		3.5		4.6
Sarafloxacin		101.0		2.1		4.3		5.9				85.1		4.7		0.9		3.7				89.5		3.5		2.9		3.2
Difloxacin		97.9		3.2		1.2		4.1				103.6		1.8		1.6		3.1				85.6		4.5		2.4		5.2
Enoxacin		96.5		3.5		3.1		3.6				108.0		5.6		3.4		1.8				91.7		4.5		3.5		3.5
Garms nitrous oxide		81.5		4.7		1.9		5.2				86.2		3.7		2.9		2.9				92.6		4.3		4.6		3.7
Hydroxymetronidazole		109.1		4.6		2.0		6.0				92.5		3.4		4.6		3.8				88.1		1.3		6.2		3.2
1-Methyl-5-nitro-1*H*-		100.9		4.3		2.3		3.3				97.7		4.7		2.6		2.5				100.1		1.9		4.1		3.3
imidazole-2-methanol																												
Metronidazole		83.5		3.9		1.8		6.5				86.2		3.6		6.3		2.8				89.8		1.6		2.1		2.0
Tinidazole		97.6		3.8		1.5		4.6				92.5		3.8		3.9		4.2				91.8		4.9		2.9		4.2
2-Dimethyl-5-nitro-1*H*-		95.7		3.2		2.8		5.9				97.7		4.6		5.2		2.4				87.4		2.3		1.1		3.6
imidazole-1-ethanol																												
2-Methyl-5-nitro-1*H*-		89.5		3.3		1.7		3.3				86.5		4.3		3.3		3.1				86.3		2.6		5.4		4.1
imidazole-1-propanol																												
monohydrochloride																												
Hydroxy-ipronidazole		100.1		4.2		0.9		3.2				90.2		3.8		2.1		2.6				90.6		1.5		2.4		2.6
Ipronidazole		91.2		6.9		1.2		5.0				92.3		3.6		3.2		2.9				100.6		1.3		2.2		3.3
Benznidazole		95.3		6.3		1.4		4.6				92.6		3.5		3.1		2.6				93.3		2.6		4.1		3.6
Fexinidazole		94.2		3.5		1.1		3.8				92.5		4.9		2.6		3.3				88.4		3.2		2.6		2.7
Panidazole		82.2		4.8		0.9		5.6				96.5		3.7		2.6		4.3				94.9		2.8		4.6		3.9
Carnidazole		93.4		5.7		2.1		3.6				86.5		3.4		3.2		2.8				95.4		3.6		3.3		4.3
Misonidazole		97.8		2.7		3.6		2.9				100.1		2.2		2.8		3.6				85.5		3.4		3.2		4.2
Chloramphenicol^*^		93.5		4.5		1.6		1.3				80.7		2.7		3.8		3.1				97.2		2.6		2.6		3.9
Florfenicol^*^		99.5		4.9		1.9		2.1				85.2		4.0		1.8		2.5				87.9		1.4		4.1		4.3
Thiamphenicol^*^		99.8		3.1		6.3		2.3				96.3		4.3		1.5		0.9				95.6		3.4		2.4		3.6

* The spiked levels of which are 0.5, 2.5, 5.0 μg/kg, respectively.

### 2.6 实际样品的测定

按本实验所建立的方法和国家标准方法,同时对购自超市的100批次畜禽肉样品(猪肉、鸡肉、牛肉、羊肉各25批次)以及4批次已知阳性样品进行检测。结果如表5所示,在购自超市的100批次样品中有3批次猪肉样品检出磺胺嘧啶(89.2、78.1、105.3 μg/kg), 2批次鸡肉样品检出沙拉沙星(56.3、102.0 μg/kg),其余样品未检出。

**表5:** 采用不同检测方法对实际样品的测定结果

Sample number	Sample	Compound	Contents/(μg/kg)		
			Original value	This method	National standard method
CSGM-05	pork	sulfadiazine	/	89.2	90.0
CSGM-13	pork	sulfadiazine	/	78.1	78.0
CSGM-18	pork	sulfadiazine	/	105.3	105.2
CSGM-01	chicken	sarafloxacin	/	56.3	56.4
CSGM-07	chicken	sarafloxacin	/	102.0	102.0
LYFC-01	chicken	metronidazole	128.2	127.8	128.1
LYFC-01	pork	ennosault	312.0	311.0	320.0
LYFC-02	pork	sarafloxacin	102.0	101.9	102.3
LYFC-01	mutton	sulfadiazine	98.5	98.4	98.6

CSGM: actual samples purchased from supermarkets; LYFC: known positive samples; /: no original value.

通过对实际样品以及部分已知阳性样品进行检测,本实验所建立方法与国家标准方法的检测结果基本一致。对两种方法的检测过程进行比较,完成上述基质及目标化合物的检测需要用到3个国家标准方法(农业部1077号公告-1-2008、GB 31658.23-2022和GB/T 21318-2007),而本实验建立的方法可实现一次样品制备,正离子源模式可同时检测38种目标化合物,负离子源模式可同时检测3种目标化合物。该方法具有简单、快捷、试剂消耗量小等优点,可被用于畜禽肉样品中41种兽药残留的定性筛查和定量分析。

## 3 结论

本研究通过对畜禽肉的基质特点以及41种兽药的物理化学性质进行分析,采用固相萃取柱的柱通过式理念,将QuEChERS净化填料与多壁碳纳米管相结合、颗粒材料去脂与溶剂去脂相结合,并对DART离子源参数进行优化,建立了一种复合式净化体系结合DART-MS/MS技术测定畜禽肉中41种兽药残留的方法。本研究建立的方法可用于畜禽肉中41种兽药残留的检测,同时对建立快速、简单、便捷的药物残留检测方法具有较高的参考价值。
